# Nursing and midwifery research activity in Arab countries from 1950 to 2017

**DOI:** 10.1186/s12913-019-4178-y

**Published:** 2019-05-28

**Authors:** Waleed M. Sweileh, Huda Abu-Saad Huijer, Samah W. Al-Jabi, Sa’ed H. Zyoud, Ansam F. Sawalha

**Affiliations:** 1 Department of Biomedical Sciences, College of Medicine and Health Sciences, An-Najah National University, Nablus, Palestine; 2 Hariri School of Nursing, American University of Beirut, Beirut, Lebanon; 3 Department of Pharmacy, College of Medicine and Health Sciences, An-Najah National University, Nablus, Palestine

**Keywords:** Nursing, Midwifery, Bibliometric analysis, Arab countries

## Abstract

**Background:**

Nursing and midwifery research activity is an important indicator of the quality of healthcare services and the status of nursing profession. The main aim of this study was to assess the research activity in nursing and midwifery field in Arab countries.

**Method:**

The current study implemented bibliometric method using Scopus database. The search strategy used country affiliation or journal name or keywords as a strategy to retrieve the required documents. The study period was from 1950 to2017. Analysis included a presentation of bibliometric indicators and VOSviewer mapping of the retrieved data.

**Result:**

2935 documents were retrieved making up less than 1% of global nursing and midwifery research output. Of the retrieved documents, 25% were published in high rank (first quartile = Q1) journals. The majority (56.7%) of the retrieved documents were published in the last five years of the study period. The retrieved documents received an average of 6.9 citations per document with an *h*-index of 47. The total number of authors who took part in publishing the retrieved documents was 10,572, giving an average of 3.6 authors per article. Jordan ranked first in research output. Researchers from Jordan took part in over than one third (1023; 34.9%) of the retrieved documents. Lebanon (35.5%) ranked first in the percentage of documents published in Q1 journals. The United Arab Emirates ranked first in the percentage (67.4%) of publications with international authors. The most active journal involved in publishing nursing research from Arab countries was *Life Science Journal* (158; 5.4%). The *University of Jordan* was the most productive institution while the *American University of Beirut* ranked first in the percentage (36.9%) of documents published in Q1 journals. Author keyword analysis and10 most cited articles showed that non-communicable diseases and nursing education were the focus of nursing research in Arab countries.

**Conclusions:**

Nursing and midwifery research activity in Arab countries has dramatically increased especially over the past five years. Despite this, nursing research is still in its infancy, lagging in quantity and quality compared to developed countries.

**Electronic supplementary material:**

The online version of this article (10.1186/s12913-019-4178-y) contains supplementary material, which is available to authorized users.

## Background

The growing role of nurses and midwives in the provision of healthcare services and the growing burden of both communicable and non-communicable diseases have created an urgent need of many trained, knowledgeable, and skilled nurses who rely on scientific evidence in their daily practice [1–5]. In response to this need of skilled nurses and midwives, many Arab countries started new nursing education programs including graduate and specialized nursing programs. The increased number in nursing programs in Arab countries helped in meeting the increasing demand for nursing profession. However, research output remains an important indicator of the progress in nursing profession and the quality of healthcare services in any country [6, 7].

Bibliometric analysis is a method used for quantitative and qualitative assessment of research activity in any field [8–11]. Bibliometric analysis is used to generate indicators of the excellence and professional development. Several bibliometric studies have been published on nursing research. For example, a very recent bibliometric study about global research output on nursing was carried out [12]. A second example, a bibliometric study about robotics used to assist or augment nursing care was published [13]. A third example is a bibliometric study carried out to address the characteristics of the research utilization literature in nursing field [14]. A fourth example is a bibliometric analysis to visualize the impact of nursing research in which the authors illustrated the importance of bibliometric methodologies to explore the richness of nursing research [15].Up to the authors’ best knowledge and based on a literature review, no bibliometric analysis of nursing and midwifery research in Arab countries had been published. Therefore, the aim of the current study was to analyze research activity in the nursing and midwifery field in Arab countries. Health policymakers, educators, clinicians, and researchers will benefit from the current bibliometric study in identifying how to direct research funding and how to allocate budgets for future development of nursing profession. Assessment of nursing research activity will motivate researchers and create a nursing research alliance in Arab countries that serves to promote nursing profession, education, research, and healthcare services in Arab countries. The current study is in line with recommendations and future strategic planning of the World Health Organizations (WHO) regarding developing nursing and midwifery research and profession in the Middle Eastern region [16].

## Method

### Database used

In the current study, SciVerse Scopus was used to retrieve the required literature. Scopus is Elsevier’s abstract and citation database launched in 2004. Scopus covers nearly 22,794 active titles from over 11,000 publishers, in life sciences, social sciences, physical sciences and health sciences [17]. The use of Scopus was based on the advantages it has over other databases which make it suitable for such studies [18]. For example, Scopus is the largest database in terms of the number of indexed journals. Scopus is 100% inclusive of Medline and larger than Web of Science [18]. Scopus database is suitable for bibliometric studies because it facilitates bibliometric analysis of the retrieved literature.

This study was carried out using bibliometric methodology, which differs from scoping reviews or systematic reviews. In bibliometric analyses, retrieved documents from a single database are analyzed [19, 20]. In contrast, systematic reviews have a specific research question to be answered using a few publications [21, 22]. For scoping reviews, it is a preliminary assessment of potential size and scope of a certain research literature, including ongoing research, and aims to identify nature and extent of research evidence [23, 24].

### Search strategy

In this study, all Arab countries recognized by the Arab league were included. There are 22 Arab countries, which differ in the number of population, income, and healthcare system [25]. However, Arab countries share a common language and, to a large extent, common history and cultural standards. The list of the 22 Arab countries recognized by the Arab league differs from those defined by World Health Organization (WHO) as Eastern Mediterranean Region (EMRO), which includes 19 Arab countries besides Iran, Afghanistan, and Pakistan. The list of Arab countries includes Egypt, Kingdom of Saudi Arabia, United Arab Emirates, Qatar, Kuwait, Bahrain, Yemen, Jordan, Palestine, Syria, Iraq, Palestine, Lebanon, Tunisia, Morocco, Algeria, Sudan, Somalia, Mauritania, Djibouti, and Comoros.

In the current study, nursing research activity was defined as documents that fit one of the following three criteria. First, any document published from any school/college/faculty/department of nursing in any Arab country. Second, any document published in any nursing/midwifery journal with at least one author affiliated with any Arab country. Third, any document published in any journal and has a nursing-related keyword in the title, with at least one author affiliated with any Arab country. Nursing keywords used in the third criterion include “nursing”, “nurse”, “midwife”, “midwives”, “birth attendant*”, and “childbirth* at home”. The third criterion was used to minimize false-negative results that might be missed by the first and second criteria. The keywords used were those derived from the word nursing or midwife. The study period was from January 01, 1950 to December 31, 2017. Data analysis was performed on the same day to avoid errors due to citation dynamics with time. No approval of the ethics committee was sought for the current study since no humans or human materials were involved. The search strategy with the keywords used is shown in Additional file 1.

### The validity of the search strategy

In the current study, the research strategy was validated using methods stated in the previously published bibliometric studies [26–30]. The absence of false-negative results was confirmed by comparing two different methods of data collection; data obtained by the search strategy were compared with data available in Scopus for the selected active authors. For example, suppose that one of the active authors was Professor X and that the number of documents assigned to this author was 50 based on the bibliometric strategy. We searched Scopus for the number of documents of X using author search methodology. We did the same for the remaining authors in the top ten active list and the numbers obtained by the two methods were compared. Interclass correlation was used to assess the extent of agreement between the two methods [31–35]. The list of active authors used to test for validity was shown in Additional file 2. An excellent agreement between the two methods with an interclass correlation above 95% and a *p*-value less than 5% indicates a high validity of the search strategy. In the current study, the interclass correlation was 97% and *p*-value was < 0.01.Two of the co-authors, S.Z and A.S, carried out the validation process. A third author (W.S) intervened whenever there was disagreement between the two authors regarding any document.

For the absence of false-positive results, three authors (S.Z, S.A and A.S) reviewed the title and the abstract of the top cited 500 documents and excluded any false-positive results. Some false-positive results include documents about plants and cell cultures as well as nursing animals. The total number of false-positive results removed was 31 articles.

### Bibliometric indicators and mapping

In the current study, both quantitative and qualitative indicators were presented. Quantitative indicators included types of documents, languages encountered in the retrieved literature, annual growth pattern, ten most active countriesstratified by Gross Domestic Product (GDP) per capita [36], ten most active journals, ten most active authors,ten most active institutions, and most frequently encountered author keywords presented as network visualization map. VOSviewer software was used for mapping [37]. This software is a free program available from Leiden University [38]. In the visualization maps, author keywordsare presented as nodes. Larger node size indicates a higher frequency of occurrence of a particular keyword.Network visualization map was also created for international collaboration. The thickness of the connecting line between any two countries in the collaboration map indicates the strength of collaboration [39]. The node size in the map is a relative presentation of the research output of the country. In VOSviewer maps, a minimum of 10 occurrences was used as a threshold. No keywords were omitted from the map.

Qualitative indicators included citation analysis, most cited documents, journal rank, and Hirsh index (*h*-index) which is an indirect measure of readership and the extent of interest of other researchers in the document(s) being cited [40–44]. For citation analysis, no exclusion of self-citations was made. The rank of each journal was obtained from SCImago Journal Rank (SJR) website [45]. The SCImago Journal raking is a publicly available portal that includes the journals and country scientific indicators developed from the information contained in the Scopus® database (Elsevier B.V.). Quartile rankings (Q) were derived for each journal according to which quartile of the SJR distribution the journal occupies for the assigned subject category. Q1 denotes the top 25% of the SJR distribution, Q2 for middle-high position (between top 50% and top 25%), Q3 middle-low position (top 75% to top 50%), and Q4 the lowest position (bottom 25% of the SJR distribution).

## Result

### Research output

The search strategy yielded 2935 journal documents. The total number of nursing and midwifery publications obtained from Scopus using the same method was 5,133,097 documents in peer-reviewed journals. Therefore, the contribution of Arab countries to nursing and midwifery field was less than 1%. One hundred and six (3.6%) research articles received funding from non-Arab funding agencies or institutions.

The retrieved documents were research articles (2664; 90.8%) and review articles (161; 5.5%). In addition, there were 57 (1.9%) letters and notes, 25 (0.9%) editorials, 13 (0.4%) full conference papers, 6 (0.2%) short surveys, and 9 (0.3%) of an unknown type at the time of analysis. The vast majority of documents were in English (2914; 99.3%).The retrieved documents were published in journals indexed within the subject areas of medicine (1394; 47.5%), nursing (1383; 47.1%), and social sciences (192; 6.5%) with the certain overlap among subject areas.

### Most frequent author keywords

**Fig. 1:**
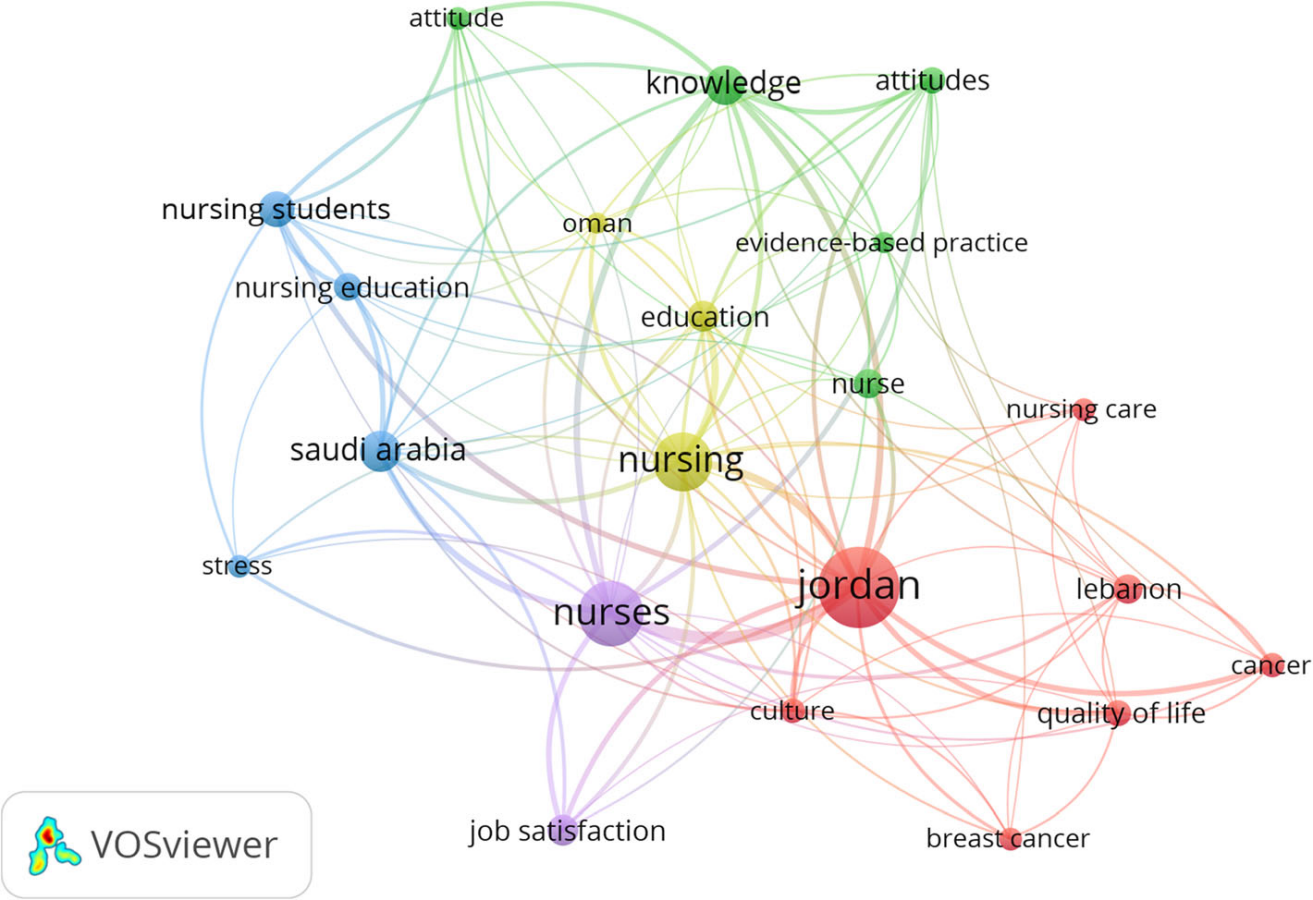
Most frequent author keywords encountered in the retrieved documents. Larger node size indicates higher frequency of occurrence

Visualization of author keywords showed that most frequent author keywords existed in five clusters (Fig. 1). The first cluster (red) included cancer-related keywords, quality of life, mental health, and Lebanon. The second cluster (green) included keywords related to knowledge, attitudes, barriers, nursing students, Saudi Arabia, and Egypt. The third cluster (light purple) included keywords related to job satisfaction of nurses. The fourth cluster (light green) included keywords such as breast cancer, prevalence, and risk factors. The fifth cluster (light blue) included keywords such as Saudi Arabia, nurses, and nursing education.

### The 10 most cited articles

The retrieved documents received 20,107 citations, including self-citations. The average number of citations was 6.9 citations per document. The *h*-index of the retrieved documents was 47. The documents that received the highest citation was a systematic review published in *BMC Geriatrics* [46].The second highly cited article was published in *The Lancet* and was about the potential role of midwifery in improving the quality of healthcare [47]. The Arab researchers who participated in this article were from Palestine and had public health affiliation. The 10 most cited articles were published in nine different journals (Table 1). The topics discussed in these documents were diverse but mainly include topics related to non-communicable diseases and nursing education.

**Table 1:** Top 10 cited articles in nursing and midwifery field published from Arab countries (1900–2017)

Title	Year	Source title	Cited by	Document Type	Country affiliation of the corresponding author	Arab country participating in the study	Is the corresponding author affiliated with a school of nursing?
Pain in elderly people with severe dementia: A systematic review of behavioural pain assessment tools [46]	2006	BMC Geriatrics	305	Review	The Netherlands	Lebanon	Yes
Midwifery and quality care: Findings from a new evidence-informed framework for maternal and newborn care [47]	2014	The Lancet	199	Review	UK	Palestine	Yes
Job stress, job performance, and social support among hospital nurses [48]	2004	Journal of Nursing Scholarship	164	Review	Jordan	Jordan	Yes
Factors associated with poor glycemic control among patients with Type 2 diabetes [49]	2010	Journal of Diabetes and its Complications	131	Article	Jordan	Jordan	Yes
Evaluating the degree of difficulty of laparoscopic colorectal surgery [50]	2008	Archives of Surgery	128	Article	Lebanon	Lebanon	No
Critical thinking in nursing education: Literature review [51]	2002	International Journal of Nursing Practice	125	Article	Saudi Arabia	Saudi Arabia	Yes
Prevalence of diabetes mellitus and its complications in a population-based sample in Al Ain, United Arab Emirates [52]	2007	Diabetes Research and Clinical Practice	107	Article	UAE	UAE	No
Factors associated with breast self-examination among Jordanian women [53]	2002	Public Health Nursing	93	Article	Jordan	Jordan	Yes
Lifetime prevalence of mental disorders in Lebanon: First onset, treatment, and exposure to war [54]	2008	PLoS Medicine	88	Article	Lebanon	Lebanon	No
IMPaCCT: Standards for paediatric palliative care in Europe [55]	2007	European Journal of Palliative Care	85	Article	UK	Lebanon	Yes

*UAE* United Arab Emirates

### Annual growth of publications

**Fig. 2:**
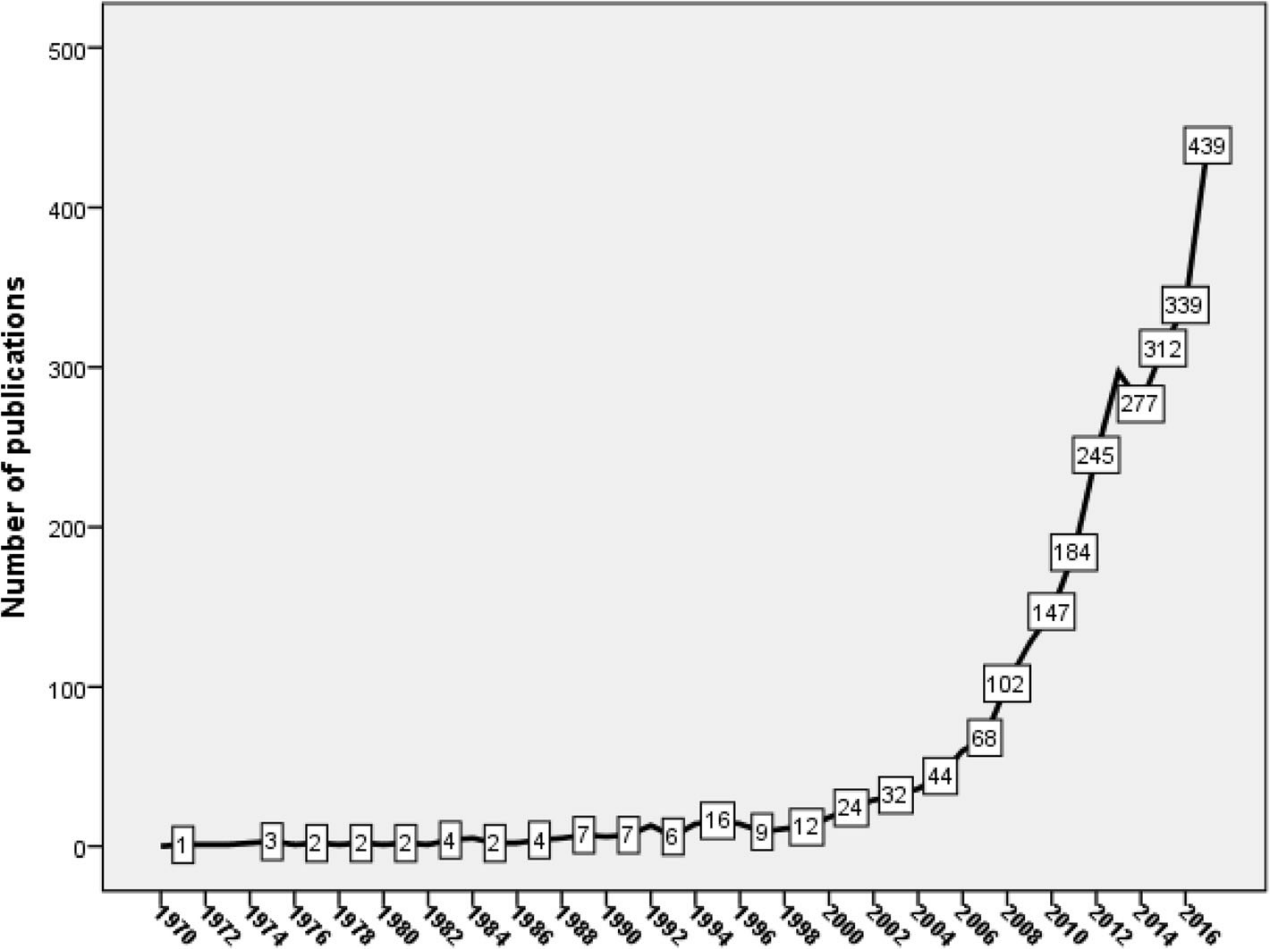
Annual growth of publications in nursing and midwifery from Arab countries

Research output was steady and low up to year 2005 followed by a noticeable upsurge (Fig. 2). The total number of documents published in the last five years of the study period was 1663(56.7%). The number of documents increased by over than three-fold in 2017 relative to that in 2008.

### The 10 most active countries

Jordan was the most active Arab country in nursing and midwifery research followed by Saudi Arabia, Egypt, and Lebanon. Researchers from Jordan contributed to over than one third (1023; 34.9%) of the retrieved documents (Table 2**)**. When research output was standardized by GDP per capita, Jordan also ranked first with 250.2 publications per GDP per capita followed by Egypt and Lebanon. Qualitatively, Lebanon ranked first in the percentage of documents published in high rank (first quartile = Q1) journals followed by Qatar and Jordan. In total, 742 (25.3%) of the retrieved documents were published in Q1 journals. When data were ranked based on *h*-index, Jordan ranked first followed by Lebanon and Kuwait.

**Table 2:** Ten most active Arab countries in nursing and midwifery research field

Country	Frequency *N* = 2935	%	GDP per capita (^a^1000)^a^	Number of publications per GDP per capita	*h*-index of the publications	Number of publications with international authors	%	Number of publications in Q1 Journals	%
Jordan	1023	34.9	4.088	250.2	34	478	46.7	293	28.6
Saudi Arabia	615	21.0	20.29	30.3	25	363	59	142	23.1
Egypt	464	15.8	3.478	133.4	22	244	52.6	68	14.7
Lebanon	315	10.7	8.25	38.2	27	196	62.3	112	35.5
Oman	153	5.2	14.981	10.2	12	90	58.9	36	23.5
Iraq	137	4.7	4.61	29.7	17	83	60.9	10	7.3
Kuwait	136	4.6	27.359	5.0	27	50	37	31	22.8
United Arab Emirates	124	4.2	37.622	3.3	14	84	67.4	32	25.8
Qatar	118	4.0	59.324	2.0	15	79	67.1	41	34.7
Palestine	64	2.2	2.943	21.7	12	17	27.3	14	21.9

^**a**^Gross Domestic Product (at purchasing power parity) per capita obtained from World Bank [56]

### International collaboration

**Fig. 3:**
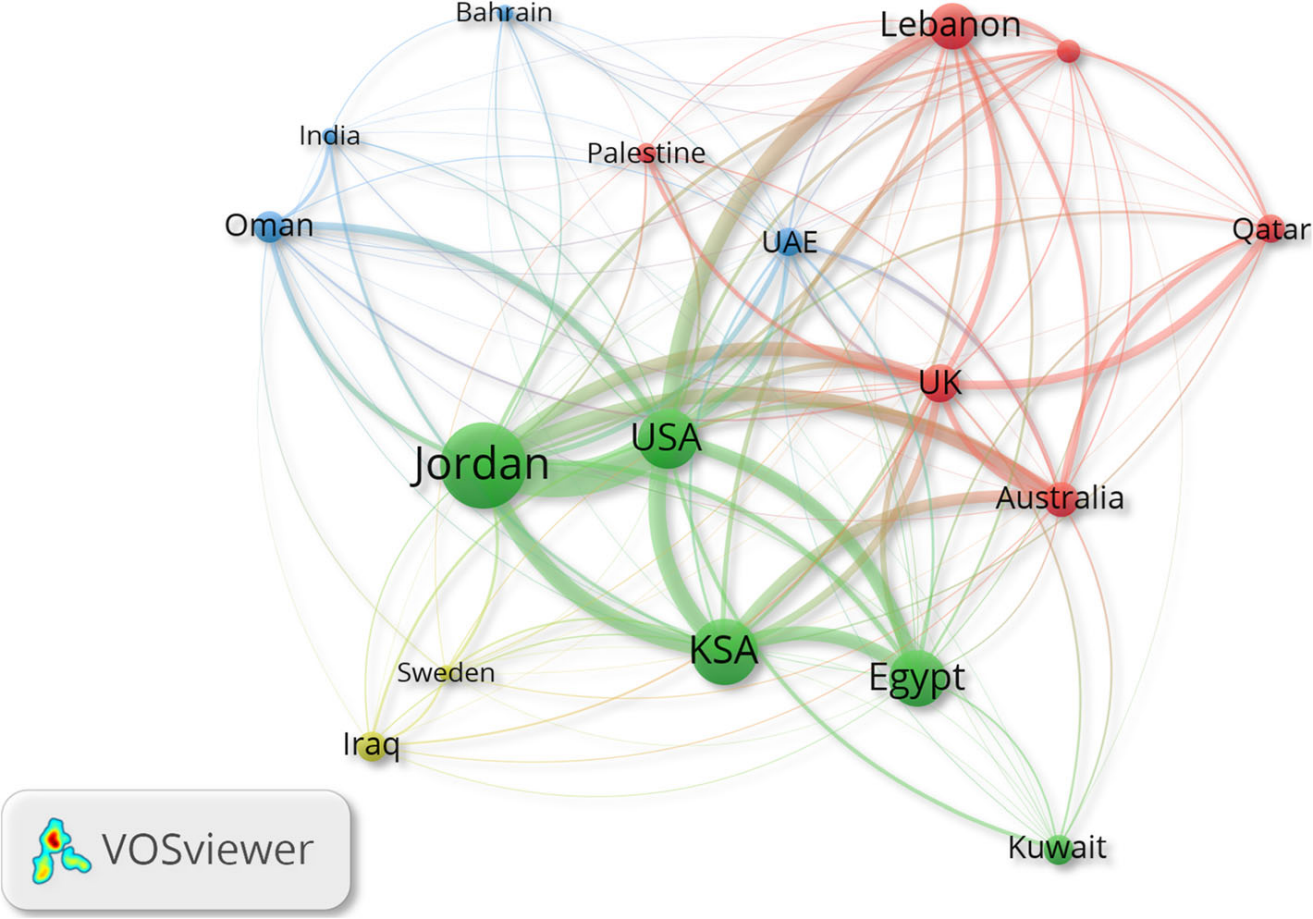
International collaboration in nursing and midwifery research in Arab countries.The thickness of the connecting line between any two countries in the collaboration map is indicative of the strength of collaboration

Analysis of international collaboration showed that United Arab Emirates ranked first in the percentage of publications (67.4%) with international authors. Qatar and Iraq ranked second and third in international collaboration. Figure 3 is a visualization of international collaboration in nursing research. The location of the USA in the map’s center indicated active research collaboration between the USA and many Arab countries. Based on the thickness of the connecting line, the strongest research collaboration was between the USA and Jordan (link strength = 200), the USA and KSA (link strength = 85), the USA and Lebanon (link strength = 84), Jordan and KSA (link strength = 82), Jordan and Australia (link strength = 67), and KSA with Egypt (link strength = 66).

### The 10 most active journals

Analysis of the retrieved documents showed that *Life Science Journal* ranked first (158, 5.4%) in publishing documents from Arab countries followed by *International Nursing review* (72; 2.5%) and *International Journal of Nursing Practice* (66; 2.2%) (Table 3). Approximately 29% of documents produced by Egyptian nursing scholars were published in *Life Science Journal*. The Jordanian scholars authored the majority (93%) of nursing documents published in the *Jordan Medical Journal*. The *Saudi Medical Journal (SMJ)* was one of the active journals. The *SMJ* is based in Saudi Arabia and Saudi scholars were the most active researchers in publishing in this journal.

**Table 3:** Ten most active journals in publishing nursing and midwifery field from researchers in Arab countries

Journal	Frequency N = 2935	%	Country publishing the journal	IF Thompson Reuters	Arab country with the highest percentage of publication in the journal
Life Science Journal	158	5.4	China	–	Egypt
International Nursing Review	72	2.5	UK	1.517	Jordan
International Journal Of Nursing Practice	66	2.2	UK	1.018	Jordan
Eastern Mediterranean Health Journal	64	2.2	Switzerland (WHO)	0.663	Egypt
Journal Of Advanced Nursing	51	1.7	UK	1.998	Jordan
Journal Of Clinical Nursing	51	1.7	UK	1.214	Jordan
Saudi Medical Journal	50	1.7	Saudi Arabia	0.73	Saudi Arabia
International Journal Of Nursing Studies	48	1.6	UK	3.755	Jordan
Jordan Medical Journal	46	1.6	Jordan	–	Jordan
Nurse Education Today	42	1.4	UK	2.533	Jordan

### Authorship analysis

In total, 10,572authors participated in publishing the retrieved documents, giving an average of 3.6 authors per article and a median of three. There were 446 (15.2%) single-authored publications.

### Ten most active institutions

Quantitative analysis showed that the *University of Jordan* ranked first (339; 11.6%) followed by the *Jordan University of Science and Technology* (299; 10.2%) and *American University of Beirut* (252; 8.6%). The ten most active list of institutions included five institutions in Jordan, one in Lebanon, one in Kuwait, one in Egypt, and one in Saudi Arabia (Table 4). No significant correlation was found between nursing research output and Webometrics or QS University ranking of the ten most active institutions. When data were stratified by percentage of documents published in Q1,the *American University of Beirut* ranked first with approximately over one third of its documents being published in Q1 journals followed by *King Saud University* and *Jordan University of Science and Technology*.

**Table 4:** Ten most active institutions in the nursing and midwifery field

Institution	Frequency N = 2935	%	Number of publications in Q1 Journals	%	Country affiliation	h-index of the publications	Webometrics University ranking in Arab world	QS University Ranking
The University of Jordan	339	11.6	87	25.7	Jordan	24	10	9
Jordan University of Science and Technology	299	10.2	91	30.4	Jordan	27	17	14
American University of Beirut	252	8.0	93	36.9	Lebanon	28	5	1
Hashemite University	186	6.3	44	23.7	Jordan	19	33	48
Alexandria University	122	4.2	20	16.4	Egypt	16	9	15
King Saud University	113	3.9	37	32.7	Saudi Arabia	19	1	3
Sultan Qaboos University	111	3.8	29	26.1	Oman	25	44	10
University of Kuwait	99	3.4	19	19.2	Kuwait	25	16	19
Al Al-Bayt University	96	3.3	28	29.2	Jordan	13	78	–
University of Mutah	85	2.9	25	29.4	Jordan	11	88	–

## Discussion

In the current study, research activity on nursing and midwifery in Arab countries was assessed and analyzed. The study showed low nursing research activity and research collaboration in Arab countries. In the past two decades, there was an increase in the number of researchers and the number of nursing faculties in Arab countries which participated in the growth of nursing and midwifery publications [57, 58]. For example, in Jordan, at least nine governmental and non-governmental faculties of nursing are running. Similar situation exists in KSA and Egypt. A second potential reason for the upsurge in the number of publications is research collaboration. Several publications resulted from collaboration between researchers in Arab countries with their mentors or colleagues or collaborators in developed countries [59–61]. The third potential reason is the academic pressure and competition which made researchers struggle to be promoted or get a tenure. In most Arab countries, academic promotion is based on the number and quality of publications. This is a strong driving force for competition among academics in various institutions to publish for tenure and promotion purposes. Finally, governments, health policy makers, international health organizations, and patients’ demand for better healthcare quality [62–67] are also potential reasons for faculties of nursing to excel and adopt international standards in nursing education, practice and research. Recently, many academic institutions in Arab countries became aware of the importance of research component in regional and international university ranking systems. This was a major driving force for some universities in Arab region to give financial incentives to researchers based on their research activity. Furthermore, more funding became available from universities to encourage researchers to publish. Unfortunately, no data are available concerning the annual volume of financial support for research activity from most active Arab universities. Therefore, correlation between funding and volume of publications from Arab universities remain an intuitive one.

The current study showed that the mean number of citations per article and the *h*-index of the retrieved documents were low when compared to other fields [68–70]. This was unsurprising given the fact that research in this field is a relatively recent. Most documents published from Arab countries in the nursing and midwifery field are of local or regional interest. Therefore, it is difficult to attract a high number of citations from international authors [71]. A third potential reason for the low mean number of citations is the lack of a nursing and midwifery journal published from the Arab region. Such a journal will create a comprehensive database for Arab scholars, which will increase the visibility of publications and citations. The low number of citations, *h*-index, and the low percentage of documents published in Q1 journals indicate modest quality of publications from Arab countries. The current study showed limited number randomized clinical trials (RCTs) in the retrieved literature. Arab countries are still behind in this type of studies because of cultural and legal issues [72, 73]. Not all Arab countries have laws that support the implementation of RCT and other types of experimental studies that involve humans [73–75]. Patients in Arab countries might not be willing to participate or volunteer in such studies. Patients in Arab countries lack the awareness of such type of studies and might think negatively and suspiciously about such type of studies [72, 74]. One final point is that researchers in Arab countries might lack the expertise or adequate funding to carry out such studies given that RCT require team work with diverse experiences as well as adequate funding to cover all aspects of the trials. A recently published Delphi survey about nursing research priorities in Middle Eastern region concluded that “critical research priorities should focus on population-based health topics” [76].

The current study showed that nursing and midwifery research in some Arab countries has made noticeable progress while research in other Arab countries is still lagging and even “neglected”. The Arab Gulf countries (Oman, Qatar, UAE, Bahrain, KSA, and Kuwait)with high income and research funding are present in the list of active countries. In the past three decades, all Arab Gulf countries established various medical colleges including ones for nursing and allied health sciences [77, 78]. Furthermore, most institutions in Arab Gulf countries had successfully recruited researchers who helped improve nursing research output from these countries [79]. Despite the huge budget available for medical education and research in Arab Gulf countries, both Jordan and Lebanon made an outstanding quantitative and qualitative contribution to nursing and midwifery research that surpassed that of Arab Gulf countries. Medical and nursing education in Lebanon started as early as 1900s while that in Jordan is relatively recent. It seems that conflict in the Middle East created a high demand for medical services in both Jordan and Lebanon, which prompted greater planning for nursing education and research.

### Limitations

Bibliometric analysis is confounded by the absence of a comprehensive database that includes all documents published in all peer-reviewed journals. Despite this, Scopus remains an ideal database to carry out such studies because it is the largest of all available databases. In bibliometric analyses, the research findings depend on the retrieved documents, which in turn depend on the implemented search strategy. False-negative results are still a possibility that might cause an underestimation of the research output and citation of a particular author, institution, country, or journal. Therefore, the results in this study need to be interpreted based on the search strategy and the database used. Finally, self-citation might affect the h-index value for authors, institutions, and countries. Therefore, the value of *h*-index is sometimes misleading when self-citation is included in the analysis.

## Conclusions

Nursing and midwifery research in Arab countries is part of the overall medical research in Arab countries, which has been lagging relative to other countries [80–89]. However, nursing and midwifery research in Arab countries is on the rise and expected to advance nursing practice and healthcare services in Arab countries. Researchers in active Arab countries such as Jordan, Lebanon, and Gulf countries need to seek high quality publications to contribute positively to the global advancement of nursing profession. Researchers in Arab countries need to launch RCTs to contribute to evidence-based nursing practice. It is also important that researchers in Arab countries start focusing on nursing topics in the field of infectious diseases and potential role of nursing staff in minimizing the burden of such diseases. Authors of the current study recommend stronger research collaboration between active Arab countries and those lagging in nursing research. Nursing conferences in Arab world need to be encouraged to help better communication among all those in the field.

## Additional files

Additional file 1: Keywords and search methodology used in Scopus to retrieve related documents. Supplement 1 included search query and a step - by - step guide to retrieve the required data from Scopus database. (DOC 39 kb)

Additional file 2: List of active researchers used to test validity of the search strategy. Supplement 2 included a list of active authors with their research output obtained by two different methods was compared to test for the validity of the search strategy. (DOC 34 kb)

## Data Availability

The datasets used and/or analysed during the current study are available from the corresponding author on reasonable request.

## Abbreviations

AUB: American University of Beirut
EMRO: Eastern Mediterranean Region
GDP: Gross Domestic Product
GDP: Gross Domestic Product
KSA: Kingdom of Saudi Arabia
Q: Quartile
RCT: Randomized Clinical Trial
SJR: SCImago Journal Rank
SMJ: Saudi Medical Journal
UAE: United Arab Emirates
UK: United Kingdom
USA: United States of America
WHO: World Health Organization
