# Supplementary material for: Nursing and midwifery research activity in Arab countries from 1950 to 2017
Source: BMC Health Serv Res. 2019 May 28;19:340. doi: 10.1186/s12913-019-4178-y (PMC6537303; doi:10.1186/s12913-019-4178-y)
Supplement: Supplementary file 1 — Keywords and search methodology used in Scopus to retrieve related documents. Supplement 1 included search query and a step - by - step guide to retrieve the required data from Scopus database. (DOC 39 kb) [file 12913_2019_4178_MOESM1_ESM.doc]

**Manuscript title: Nursing and midwifery research activity in Arab countries from 1950 to 2017**

**Supplement 1: Keywords and search methodology used in Scopus to retrieve related documents**

Waleed M. Sweileh^1,*^
Email: [waleedsweileh@yahoo.com](mailto:waleedsweileh@yahoo.com)

Huda Abu-Saad Huijer^2^

E-Mail: [huda.huijer@aub.edu.lb](mailto:huda.huijer@aub.edu.lb)

Samah W. Al-Jabi^3^
Email: [samahjabi@yahoo.com](mailto:samahjabi@yahoo.com)

Sa’ed H. Zyoud^3^

Email: [saedzyoud@yahoo.com](mailto:saedzyoud@yahoo.com)

Ansam F. Sawalha^1^
Email: [ansam@najah.edu](mailto:ansam@najah.edu)

^1^ Department of Biomedical Sciences, College of Medicine and Health Sciences, An-Najah National University, Nablus, Palestine

^2^ Hariri School of Nursing, American University of Beirut, Lebanon

^3^Department of Pharmacy, College of Medicine and Health Sciences, An-Najah National University

^*^ Corresponding author: Department of Biomedical Sciences, College of Medicine and Health Sciences, An-Najah National University, Nablus, Palestine

| **Step** | **Function** |
| --- | --- |
| **1** | AFFIL(Jordan and nursing) OR AFFIL(Egypt and nursing) OR AFFIL(Saudi and nursing) OR AFFIL(Lebanon and nursing) OR AFFIL(Arab and nursing) OR AFFIL(Oman and nursing) OR AFFIL(Kuwait and nursing) OR AFFIL(Qatar and nursing) OR AFFIL(Bahrain and nursing) OR AFFIL(Yemen and nursing) OR AFFIL(Iraq and nursing) or AFFIL(Sudan and nursing) or AFFIL(Tunisia and nursing) or AFFIL(Morocco and nursing) OR AFFIL(Algeria and nursing) OR AFFIL(Mauritania and nursing) OR AFFIL(Somalia and nursing) or AFFIL(Palestine and nursing) OR AFFIL(Syria and nursing) OR AFFIL(Libya and nursing) or AFFIL(Djibouti and nursing) or AFFIL(Comoros and nursing) |
|  | AND |
| **2** | NOT AFFIL("nursing and allied health" or "pharmacy and nursing" or "All. Hlth. Sci. and Nursing" or "dentistry and nursing")) |
|  | OR |
| **3** | (AFFILCOUNTRY ( syria* OR leban* OR palestin* OR jordan* OR egypt* OR morrocc* OR tunisi* OR iraq* OR kuwait OR "saudi*" OR yemen* OR sudan OR libya* OR algeri* OR somal* OR mauritan* OR qatar OR arab OR oman OR comoros OR djibouti OR bahrain ) AND TITLE ( nurse* OR nursing OR midwif* ) |
|  | AND |
| **4** | NOT TITLE ( nursery OR "nursing mother*" OR "nursing home*" ) AND NOT TITLE-ABS ( animal OR veter* OR plant OR agricult* ) AND NOT SRCTITLE ( peptid* OR mycotox* ) AND NOT SRCTITLE ( peptid* OR mycotox* OR animal OR plant OR agriculture OR ruminant OR ecology OR animal OR plant OR agriculture OR ruminant OR ecology OR information OR computer OR veterinary OR computing OR communication )) |
|  | OR |
| **5** | (AFFILCOUNTRY ( syria* OR leban* OR palestin* OR jordan* OR egypt* OR morrocc* OR tunisi* OR iraq* OR kuwait OR "saudi*" OR yemen* OR sudan OR libya* OR algeri* OR somal* OR mauritan* OR qatar OR arab OR oman OR comoros OR djibouti OR bahrain ) AND srcTITLE ( nurse* OR nursing OR midwif* ))) and AFFILCOUNTRY ( syria* OR leban* OR palestin* OR jordan* OR egypt* OR morrocc* OR tunisi* OR iraq* OR kuwait OR "saudi*" OR yemen* OR sudan OR libya* OR algeri* OR somal* OR mauritan* OR qatar OR arab OR oman OR comoros OR djibouti OR bahrain )) |
|  | OR |
| **6** | or (AFFILCOUNTRY ( syria* OR leban* OR palestin* OR jordan* OR egypt* OR morrocc* OR tunisi* OR iraq* OR kuwait OR "saudi*" OR yemen* OR sudan OR libya* OR algeri* OR somal* OR mauritan* OR qatar OR arab OR oman OR comoros OR djibouti OR bahrain ) and affil(NURSING) and title-abs(NURSING OR MIDWIFERY)) ) |
|  | AND |
| **7** | AND ( LIMIT-TO ( SRCTYPE,"j" ) ) AND ( EXCLUDE ( DOCTYPE,"er" ) ) AND ( EXCLUDE ( PUBYEAR,2018) ) |
