# Supplementary material for: Nursing and midwifery research activity in Arab countries from 1950 to 2017
Source: BMC Health Serv Res. 2019 May 28;19:340. doi: 10.1186/s12913-019-4178-y (PMC6537303; doi:10.1186/s12913-019-4178-y)
Supplement: Supplementary file 2 — List of active researchers used to test validity of the search strategy. Supplement 2 included a list of active authors with their research output obtained by two different methods was compared to test for the validity of the search strategy. (DOC 34 kb) [file 12913_2019_4178_MOESM2_ESM.doc]

**Supplement 2**

List of active researchers used to test validity of the search strategy

| **Author** | **Frequency**  **N**  **Obtained by Scopus search strategy** | **Frequency**  **Obtained by manual search strategy***** | **Affiliation** |
| --- | --- | --- | --- |
| Abu-Saad, H.H. | 62 | 63 | American University of Beirut, Hariri School of Nursing, Beirut, Lebanon |
| Hamdan-Mansour, A.M. | 53 | 53 | Department of Community Health Nursing, The University of Jordan, Amman, Jordan |
| Ahmad, M.M. | 40 | 39 | The University of Jordan, Clinical Nursing Department, Amman, Jordan |
| Darawad, M.W. | 32 | 33 | The University of Jordan, School of Nursing, Amman, Jordan |
| Mrayyan, M.T. | 30 | 30 | Hashemite University, Faculty of Nursing, Zarqa, Jordan |
| Khader, Y.S.* | 30 | 31 | Jordan University of Science and Technology, Department of Community Medicine, Public Health and Family Medicine, Irbid, Jordan* |
| Noureddine, S. | 30 | 30 | American University of Beirut, Hariri School of Nursing, Beirut, Lebanon |
| Muliira, J.K. | 26 | 27 | Sultan Qaboos University, Department of Adult Health and Critical Care Nursing, Muscat, Oman |
| Hameed, I.H.** | 25 | 25 | University of Babylon, College of Nursing, Babylon, Iraq** |
| Al-Hussami, M. | 23 | 23 | The University of Jordan, School of Nursing, Amman, Jordan |

*This author is not affiliated with faculty of nursing but has extensive research collaboration with faculties of nursing

**This author is in the college of nursing but his field is biotechnology, molecular biology, microbiology

***Correlation between the two sets of data was > 95%
